# Antigen-presenting B cells promote TCF-1^+^ PD1^-^ stem-like CD8^+^ T-cell proliferation in glioblastoma

**DOI:** 10.3389/fimmu.2023.1295218

**Published:** 2024-01-10

**Authors:** David Hou, Hanxiao Wan, Joshua L. Katz, Si Wang, Brandyn A. Castro, Gustavo I. Vazquez-Cervantes, Victor A. Arrieta, Silpol Dhiantravan, Hinda Najem, Aida Rashidi, Tzu-yi Chia, Tarlan Arjmandi, Jimena Collado, Leah Billingham, Aurora Lopez-Rosas, Yu Han, Adam M. Sonabend, Amy B. Heimberger, Peng Zhang, Jason Miska, Catalina Lee-Chang

**Affiliations:** ^1^ Department of Neurological Surgery, Feinberg School of Medicine, Northwestern University, Chicago, IL, United States; ^2^ Feinberg School of Medicine, Northwestern University, Chicago, IL, United States; ^3^ Department of Neurological Surgery, University of Illinois Chicago, Chicago, IL, United States; ^4^ Department of Biotechnology, McCormick School of Engineering, Northwestern University, Evanston, IL, United States; ^5^ Lou and Jean Malnati Brain Tumor Institute, Chicago, IL, United States

**Keywords:** GBM, B cells, stem-like memory CD8^+^ T cell, immunological synapse, anti-tumor response

## Abstract

Understanding the spatial relationship and functional interaction of immune cells in glioblastoma (GBM) is critical for developing new therapeutics that overcome the highly immunosuppressive tumor microenvironment. Our study showed that B and T cells form clusters within the GBM microenvironment within a 15-μm radius, suggesting that B and T cells could form immune synapses within the GBM. However, GBM-infiltrating B cells suppress the activation of CD8^+^ T cells. To overcome this immunosuppression, we leveraged B-cell functions by activating them with CD40 agonism, IFNγ, and BAFF to generate a potent antigen-presenting B cells named B_Vax_. B_Vax_ had improved antigen cross-presentation potential compared to naïve B cells and were primed to use the IL15-IL15Ra mechanism to enhance T cell activation. Compared to naïve B cells, B_Vax_ could improve CD8 T cell activation and proliferation. Compared to dendritic cells (DCs), which are the current gold standard professional antigen-presenting cell, B_Vax_ promoted highly proliferative T cells *in-vitro* that had a stem-like memory T cell phenotype characterized by CD62L^+^CD44^-^ expression, high TCF-1 expression, and low PD-1 and granzyme B expression. Adoptive transfer of B_Vax_-activated CD8**^+^** T cells into tumor-bearing brains led to T cell reactivation with higher TCF-1 expression and elevated granzyme B production compared to DC-activated CD8**^+^** T cells. Adoptive transfer of B_Vax_ into an irradiated immunocompetent tumor-bearing host promoted more CD8^+^ T cell proliferation than adoptive transfer of DCs. Moreover, highly proliferative CD8**^+^** T cells in the B_Vax_ group had less PD-1 expression than those highly proliferative CD8**^+^** T cells in the DC group. The findings of this study suggest that B_Vax_ and DC could generate distinctive CD8**^+^** T cells, which potentially serve multiple purposes in cellular vaccine development.

## Introduction

The past decade of immunotherapeutic development has seen many significant strides, with immune checkpoint blockade becoming part of the standard of care for many tumor types and cellular therapies entering the clinic. However, in glioblastoma (GBM), many challenges still exist for the successful clinical translation of immunotherapies. One significant roadblock is the harsh immunosuppressive tumor microenvironment (TME) that blocks anti-tumor immune responses (1, 2). Thus, understanding how different cells spatially relate to one another allows for an improved understanding of cell-to-cell interactions and function within the TME. In brain tumors, these cellular interactions are key in driving TME-mediated tumor progression and therapeutic resistance (3, 4).

**Figure 1:**
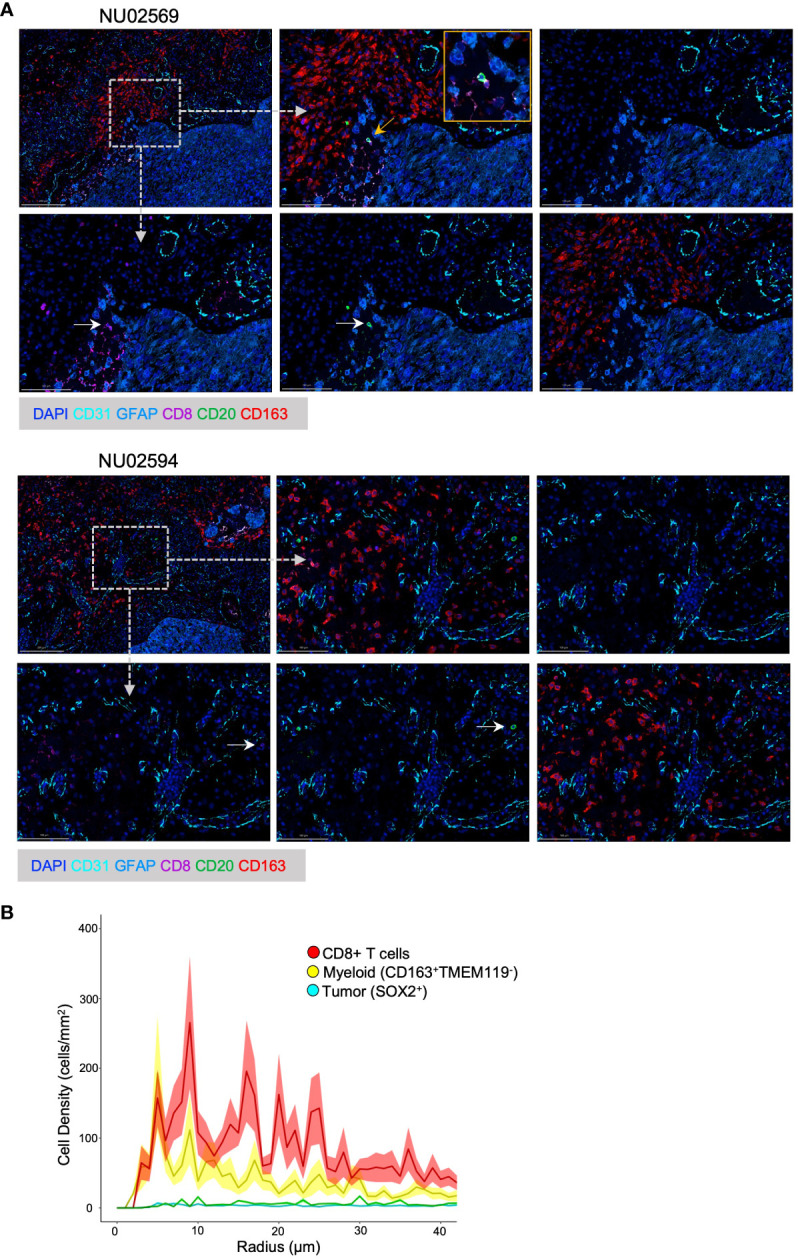
Spatial analysis of B T and myeloid cells in GBM TME. **(A)** Representative Spatial Multiplex seqIF™ images generated using the COMET™ platform (Lunaphore Technologies) showing cellular infiltration in the GBM TME from patient NU02569 and NU02594. The TME was analyzed for prevalence of B cells (CD20^+^), cytotoxic T cells (CD8^+^), myeloid cells (CD163^+^), epithelial cells (CD31^+^), tumor cells and astrocytes (GFAP^+^) combined with DAPI staining. For each patient, upper images from left to right: low magnification of full panel, high magnification of the full panel, high magnification background stain (DAPI, GFAP and CD31). Lower images from left to right: High magnification of CD8^+^, CD20^+^, CD163^+^ cells. White arrows highlight CD8^+^ or CD20^+^ cells. Yellow arrow and box mark the magnified region. Dashed arrows and boxes mark the regions of the high magnification images. **(B)** Paired correlation analysis (n=21) of density of CD8^+^ T cells, myeloid cells (CD163^+^ TMEM119^-^), and tumor cells (SOX2^+^) based on radial distance from the nearest B-cell from images obtained with Vectra 3 Automated Quantitative Pathology Imaging System.

Spatial relationships are important in understanding the TME because they provide context for interpreting cellular function. Our previous work focused on studying the role of B cells in GBM, and we show that despite B cell exclusion in GBM (5), they can play both a suppressive and pro-inflammatory role in brain tumors. For example, myeloid cells can hijack B cells using microvesicles and turn them into highly suppressive regulatory B cells (5). However, we recently developed a B cell-based cellular vaccine (B_Vax_) (**Figure 1A**) utilizing highly activated, 4-1BBL^+^ B cells that could overcome TME immunosuppression and elicit a strong T cell response against tumors in our preclinical GBM models (6). B_Vax_ therapy demonstrated enhanced CD8^+^ T cell expansion and durability in GBM, resulting in improved anti-tumor response when juxtaposed with DCs Field (6), a popular therapeutic approach for anti-tumor vaccine development (7, 8).

Different subsets of CD8**^+^** T cells have been shown to have different efficacies in mounting anti-tumor immune responses (9, 10). Activated T cells can be viewed as 2 large groups: effector T cells and memory T cells. While the exact mechanism of T cell differentiation into effector or memory subsets remains unknown, factors such as different antigen-presenting cells with varying co-stimulation and cytokines play important roles (10–13). For example, studies have shown that IL2 controls acute expansion and differentiation of antigen-specific CD8**^+^** T cells, whereas IL7 and IL15 are key cytokines to maintain memory of CD8**^+^** T cells long term. Different types of professional antigen-presenting cells (APCs) likely have different profiles of CD8 activation based on the production of different cytokines and possibly the presentation of different types of antigens.

Stem-like memory T cells are a subset of memory T cells associated with chronic infections that can self-renew and reconstitute the entire spectrum of memory and effector T cell subsets (14–17). These stem-like memory T cells can be characterized as TCF1^+^ (18), CD62L, and CD44 expression (19). Generating stem-like memory CD8**^+^** T cells is important for immunotherapy as it promotes treatment persistence and protection against tumor recurrence (20).

While our previous work showed increased tumor-infiltrating CD8^+^ T cells and anti-tumor activity with B_Vax_ therapy, the mechanism remained unclear. In this study, we first investigated the spatial dynamics of B and CD8**^+^** T cells in GBM. We then characterized the antigen-presentation ability of our B_Vax_ compared to naïve B cells. Finally, we studied the ability of B_Vax_ to generate different CD8**^+^** T cell subsets both *in-vitro* and *in-vivo*. Our work provides insights into new strategies for maximizing anti-tumor T-cell responses.

## Materials and methods

### Human samples

The Nervous System Tumor Bank at Northwestern University was used to collect all human samples. Approval for collection was granted under the institutional review board protocol number STU00202003 and the study was conducted following the U.S. Common Rule of ethical standards. All patients signed written consent forms. Collected samples included tumor, peripheral blood, and frozen tissue from GBM patients with at least 50% tumor cellularity, as determined by neuropathologist review of H&E sections.

GBM patient survival data was analyzed from The Cancer Genome Atlas Glioblastoma Multiforme Study dataset (TCGA-GBM) using the RNA-seq platform. Patients all had stage IV GBM and were not stratified based on histology, sex, IDH status, methylation, or recurrence.

### Mice

C57BL/6, Nur77-GFP, B6 CD45.1, and OT1 were all purchased from The Jackson Laboratory and bred for use in experiments. Studies were initiated when the mice were 6-8 weeks old. Approval for all animal experimental protocols was approved by the Institutional Animal Care and Use Committee at Northwestern University under the protocol number ISO16696. All animals were housed at the Center for Comparative Medicine at Northwestern University in a dedicated pathogen-free animal facility with 12-hour light/12-hour dark cycles and ad libitum access to food and water.

### Cell lines

CT2A cells were obtained from Millipore (Sigma Aldrich). Cells were cultured in Dulbecco’s modified Eagle’s medium (Corning) supplemented with 10% fetal bovine serum (FBS; Hyclone), 100 U/ml penicillin (Corning), and 100 mg/ml streptomycin (Corning) and incubated at 37°C in 5% CO_2_. Every 2 months, cell lines were tested for *Mycoplasma* contamination using the Universal Mycoplasma Detection Kit (ATCC 30-1012K).

### Intracranial tumor implantations

Each mouse was implanted with 1x10^5^ tumor cells in a total volume of 2.5 ul of PBS. Mice were anesthetized with ketamine (100 mg/kg) and xylazine (10 mg/kg) via intraperitoneal injection. After shaving the surgical site and disinfection with povidone-iodine and 70% ethanol, an incision was made at the midline to access the skull. A 1 mm-diameter burr hole was drilled 2 mm posterior to coronal suture and 2 mm lateral to the sagittal suture. Injections were performed using a Hamilton syringe fitted with a 26-gauge blunt needle at a depth of 3.5 mm. The implantation site was then sutured closed.

### GBM sample IHC

Formalin-fixed paraffin-embedded tumor blocks of 60 GBM patients were double-labeled with rabbit anti-human CD20 (1:2 dilution, clone EP459Y, Thermo Fischer) and mouse anti-human CD8 (1:100 dilution, clone 4B11, Leica). Anti-rabbit and anti-mouse HRP secondary antibodies (both from Abcam) were used at 1:500 dilution. Twenty consecutive high-power fields were evaluated on each tumor by a board-certified neuropathologist from the Neurosurgical Department at Northwestern University. Images were captured on a Leica DMi8 inverted microscope.

### Multiplex sequential immunofluorescence (seqIF™) staining using Lunaphore COMET™

The multiplex panel included the following unconjugated antibodies: CD31 (endothelial cells; Abcam EPR3131), GFAP (Glioma tumor cells and astrocytes; Abcam EPR1034Y), CD4 (T helper cells; Abcam EPR17259), CD8 (cytotoxic T cells; Leica 4B11), CD20 (B cells; Dako Agilent L26), CD163 (macrophage scavenger receptor; Abcam EPR19518). All antibodies were validated using conventional immunohistochemistry and/or immunofluorescence (IF) staining, in conjunction with the corresponding fluorophore and the spectral 4’,6-diamidino-2-pheynlindole (DAPI; ThermoFisher Scientific) counterstain. For optimal concentration and the best signal/noise ratio, all antibodies were tested at three different dilutions, starting with the manufacturer-recommended dilution (MRD), then MRD/2 and MRD/4. Secondary Alexa Fluor 555 (ThermoFisher Scientific) and Alexa Fluor 647 (ThermoFisher Scientific) were used at 1/200 and 1/400 dilutions, respectively. The optimizations and full runs of the multiplex panel were executed using the sequential IF (seqIF™) methodology integrated into the Lunaphore COMET™ platform (characterization 2 and 3 protocols and seqIF™ protocols, respectively). The staining can be performed at a maximum of 4 tissue slides simultaneously following automated cycles of 2 antibodies’ staining at a time, followed by imaging and elution, where no sample manipulation is required (21). All reagents were diluted in Multistaining Buffer (BU06, Lunaphore Technologies). The elution step lasted 2 minutes for each cycle and was performed with Elution Buffer (BU07-L, Lunaphore Technologies) at 37°C. Quenching lasted for 30sec and was performed with Quenching Buffer (BU08-L, Lunaphore Technologies). Antibody incubation time was set to 4min for all primary and secondary antibodies to 2min. Imaging was performed with Imaging Buffer (BU09, Lunaphore Technologies) and an integrated epifluorescent microscope at 20x magnification using DAPI, TRITC, and Cy5 channels. Image registration was performed immediately after concluding the staining and imaging procedures by COMET™ Control Software. Each seqIF™ protocol resulted in a multi-layer OME-TIFF file where the imaging outputs from each cycle were stitched and aligned. COMET™ OME-TIFF files contain a DAPI image, intrinsic tissue autofluorescence in TRITC and Cy5 channels, and a single fluorescent layer per marker. Markers were subsequently pseudocolored for visualization of markers in the Viewer from Lunaphore.

### Multiplex immunofluorescence staining

GBM sections of 5μm thickness were obtained from FFPE embedded tumor tissue of 21 different patients. Deparaffinization of the slides was achieved using xylene followed by rehydration in histological grade ethanol and fixed with 3% hydrogen peroxide in methanol before heat-induced epitope retrieval using BOND epitope retrieval solution (pH6) or pH9 EDTA buffer for 20 minutes. 3,3’-diaminobenzidine chromogen staining was initially performed to determine the optimal concentrations of each antibody in human GBM tissues. Primary antibodies were diluted with 1x Opal Antibody diluent/block solution and were used in the following order coupled with the indicated Opal dyes: 1) TMEM119 (cat HPA051870, Sigma-Aldrich, 1:250 dilution) with Opal 520 (1:100 dilution), 2) CD163 (cat ab213612, clone EPR19518, Abcam, 1:600 dilution) with Opal 570 (1:800 dilution), 3) SOX2 (cat ab92494, clone EPR3131, Abcam, 1:5000 dilution) with Opal 620 (1:150 dilution), 4) CD20cy (cat M075501-2, clone L26, Agilent Dako, 1:400 dilution) with Opal 540 (1: 200 dilution), 5) CD8 (cat CD8-4B11-L-CE, clone 4B11, Leica Biosystems, RTU) with Opal 650 (1:100 dilution). Multiplex staining was performed in multiple cycles involving a heat-induced epitope retrieval step, protein blocking, epitope labeling, and signal amplification. Once all markers were stained, spectral DAPI was used to counterstain the slides and were mounted using Prolong Diamond Antifade Mountant.

### Imaging and analysis of multispectral images

Multispectral imaging (MSI) was performed using the Vectra 3 Automated Quantitative Pathology Imaging System from Akoya Biosciences. First, whole slide images were acquired after auto-adjusting focus and signal intensity. Then, MSI was acquired from the tumor regions delineated by a certified neuropathologist at 20x of the original magnification. For analysis of MSI, we created a spectral library for all Opal dyes to subject acquired multispectral images to spectral unmixing that enabled the identification and separation of weakly expressing and overlapping signals from background to visualize the signal of each marker (SOX2, CD20cy, TMEM119, CD163, CD8, DAPI) in inForm Tissue Finder software (inForm 2.6, Akoya Biosciences). Using InForm, the adaptive cell segmentation feature was used to identify the analyzed cells’ nuclei and determine each cell’s nuclear and cytoplasmic compartments. A machine-learning algorithm within inForm was used in which cells were automatically assigned to a specific phenotype (SOX2^+^, TMEM119^+^, CD163^+^, CD20^+^, CD8^+^). Batch analysis was used to analyze all tumor samples under the same segmentation and phenotype settings. The processing and analysis of images from all tumor samples were exported to cell segmentation tables. Exported files from inForm were processed in R using R packages Phenoptr and PhenoptrReports to merge and create consolidated single files for each tumor sample. Consolidated files had triple cell phenotypes as outputs that we employed for further quantification and spatial analyses using the Phenoptr R addin.

### Paired correlation analysis on multiplex immunofluorescence samples

After phenotyping the multiplex immunofluorescence data, the spatstat R package1 was used to calculate the pair correlation functions (PCFs) between B cells and tumor-associated macrophages (TAMs), tumor cells, T cells, and microglia. B cells were defined as CD20^+^, TAMs as CD163^+^ TMEM119^-^, tumor cells as SOX2^+^, and T cells as CD8^+^. Raw PCFs were calculated for each sample and averaged across all samples. Raw PCFs and standard deviations were plotted for a maximum radius of 50 microns (22).

### B_Vax_ generation

B_Vax_ were generated following the materials and methods outlined in Hou and Katz et al. (23) and Lee-Chang et al. (6). Briefly, spleens and lymph nodes were harvested from CT2A tumor-bearing or SIINFEKL-immunized mice, and 4-1BBL^+^ B cells were isolated and cultured with anti-CD40, BAFF, and IFNγ in complete RPMI media (cRPMI, RPMI 1640 supplemented with sodium pyruvate, MEM amino acids, HEPES, 2-mercaptoethanol, and penicillin/streptomycin) to generate B_Vax_. B_Vax_ were then pulsed with either tumor lysate or SIINFEKL peptide for at least 4 hours before use for downstream assays. For more details, please refer to Hou and Katz et al. and Lee-Chang et al. (23).

### Dendritic cell generation

Bone marrow was isolated from the femur of tumor-bearing mice, and RBCs were lysed using ACK lysing buffer (Thermo Fisher A1049201). The remaining cells were washed in cRPMI and cultured in cRPMI supplemented with GM-CSF (Peprotech 315-03, 40ng/ml) and IL4 (Peprotech 214-14, 40ng/ml) for 3 days at 37°C. On the third day, media was replaced with fresh cRPMI supplemented with the same concentration of GM-CSF and IL4 and cultured for another 3 days. DCs were then pulsed with either tumor lysate or SIINFEKL peptide for at least 4 hours before use for downstream assays.

### CD8^+^ T cell isolation and *in-vitro* APC coculture

CD8**^+^** T cells were isolated from spleens and lymph nodes from CT2A tumor-bearing OT-1 or C57BL/6 mice (STEMCELL, 19858). CD8**^+^** T cells were co-cultured with antigen-presenting cells at 4:1 ratio for 3 days. To measure the proliferation cycles, CD8**^+^** T cells were labeled with CellTrace on Day 0 (ThermoFisher, C34557). A suboptimal concentration of IL15 (6.5ng/ml) was added in the initial co-culture and 48 hours after. IL15 concentration was determined based on cell viability, CD62L, CD44, and PD1 expression (**Supplementary Figures 1A–C**). CD8**^+^** T cell proliferation was analyzed by flow cytometry (BD FACS Symphony analyzer and analyzed with FlowJo software) 72 hours after the initiation of the experiment.

### TCR sequencing

After 72 hours of co-culture as previously described, cells were washed twice with PBS. TILs were magnetically isolated using CD8-biotin (clone 17A2, BioLegend) and anti-biotin Microbeads (Miltenyi Biotec). RNA isolation from the co-culture or TILs was performed using TRIzol (Thermo Fisher Scientific). Chloroform (0.2ml) was added to the TRIzol samples. The top layer containing the RNA was precipitated with 70% isopropanol. The resulting pellets were dried and resuspended in sterile water. TCR sequencing and bioinformatic analysis were performed by Adaptive Biotechnologies using the ImmunoSEQ platform.

### Bulk RNA sequencing and analysis

Three days after coculture of CD8^+^ T cells and APCs, CD8^+^ T cells were positively selected using biotinylated CD8β antibody (Biolegend) and anti-biotin microbeads (Miltenyi Biotec). RNA from these samples was isolated using TRIzol (Thermo Fisher Scientific) to purify. Chloroform (0.2ml) was added to the TRIzol samples. The top layer containing the RNA was precipitated with 70% isopropanol. The resulting pellets were dried, resuspended in sterile water, and sent to Novogene for further processing and analysis. Novogene assessed the RNA for quality and provided all data as total counts and fragments per kilobase per million reads (FPKM).

### Whole-brain radiation and *in-vivo* CD8^+^ T cell and APC adoptive transfer

CD45.1 mice were anesthetized and intracranially implanted with 10^5^ CT2A cells using a cannula system. The cannula system included a 26-gauge guide cannula implanted 2 mm deep from the skull, further glued around the burr hole to secure the position. Mice received brain radiation of 9 Gy on Day 8 (fractionated three times 3 Gy; Gammacell 40 Exactor). Eleven days post-tumor implantation, 10^6^ APCs and 4x10^6^ CD8**^+^** T cells were co-implanted intracranially via a cannula system using a 33-gauge sterile syringe, as described previously (6).

### *In vitro* APC function

The protein uptake and presentation capability were tested as previously described. B_Vax_ or B_Naive_ was fluorescently labeled with CellTracker red CMPTX (Molecular Probes; Life Technologies) and pulsed with 15 µg/ml AF488-OVA (Molecular Probes; Life Technologies) for 30 min in complete RPMI. Excessive proteins were washed away, and cells were visualized with a Leica DMi8 microscope and analyzed using ImageJ. The antigen presentation ability was measured by anti-H-2K^b^ antibody measured by flow cytometry. The ability to expand antigen-specific CD8 T cells of OVA-pulsed B_Vax_, B_Naive,_ and DC was tested with splenic OT-1 CD8 T cells labeled with CellTrace Violet (Thermo Fisher, C34557) cultured with exogenous IL15 supplementation (6.25 ng/mL, Peprotech 210-15).

### Flow cytometry and immunophenotype analysis

Immunophenotype analysis of immune cells from tumor-bearing mice was performed at time points defined in the results section. After collecting single-cell suspensions, cells were counted and washed with staining buffer (5% bovine serum albumin, 0.001% sodium azide in PBS). Cells were incubated with 1uL Fc receptor blocking Ab (anti-CD16/32, clone 93, Biolegend) per million cells in 100uL staining buffer for 5 minutes at room temperature. For surface staining, cells were incubated with 1µL antibody per million cells for 30 minutes at 4°C. Cells were stained with Fixable Viability Dye eFluor 780 (eBioscience, Thermo Fisher) for 30 minutes at 4°C. Cells were washed twice with staining buffer. Cells were fixed and permeabilized for intracellular staining using the eBioscience Foxp3/Transcription Factor Staining Buffer Set (Invitrogen, Thermo Fisher) for 90 minutes at room temperature. Cells were washed twice with the permeabilization buffer included with the kit and incubated with 1µL antibody for 1 hour at 4°C. Data was acquired with BD FACS Symphony analyzer and analyzed with FlowJo software. Dead cells and debris were excluded using the Live/Dead staining (Viability Dye eFluor 780). B-cells were identified as CD45^+^CD11b^-^CD19^+^ cells.

### Statistical analysis

Data are shown as mean ± standard deviation for continuous variables and numbers for categorical variables. Measurements were all taken from distinct samples. Differences between 2 groups were analyzed using the Student’s t-test. Multiple groups were analyzed using one-way ANOVA with a *post hoc* Tukey’s multiple comparisons test. Animal survival curves were analyzed and generated using the Kaplan-Meier method and compared by log-rank test. Categorical variables were analyzed using Fisher’s exact tests or X^2^ tests as appropriate. All tests are two-sided with p values or Benjamini-Hochberg adjusted false discovery rates of <0.05 were considered significant. Statistical analyses were performed using GraphPad Prism 9.4.1. ns= p>0.05, * = p<0.05, ** = p<0.01, *** = p<0.001, **** = p<0.0001.

## Results

### B cells and CD8^+^ T cells colocalize in the GBM TME

We had previously shown that B and T cells can be found colocalized in the perivascular zones of the TME and that B cells inhibit T cell function (5). Using the Lunaphore COMET™ spatial multiplex platform, we further evaluated the immune spatial landscape in 2 GBM patients by staining for B cells (CD20^+^), T cells (CD8^+^), myeloid cells (CD163^+^), epithelial cells (CD31^+^), tumor cells and astrocytes (GFAP^+^). B cells and T cells could be visualized in the peritumoral areas, and the two cell types can be seen colocalized in the same zones with direct contact between B and T cells (**Figure 1A**, top row). Furthermore, myeloid cells surround lymphocyte-rich areas (**Figure 1A**, bottom row).

To further quantify the spatial relationships between B, T, and myeloid cells, we performed an unbiased paired correlation analysis on 21 patient samples to characterize cell type based on the radial distance from B cells. Our data showed that T cells had the highest cell density across a wide range of radial distances from B cells, especially at around 10 microns (**Figure 1B**). This is important because 15 μm has been shown to be the maximum distance in which an immune synapse can still form (24). Thus, we show that B and T cells can share the same spatial neighborhood in the GBM environment and are close enough to form immune synapses, suggesting that B cells can be used to modulate T cell anti-tumor immunity.

### B_Vax_ are potent antigen-presenting cells

**Figure 2:**
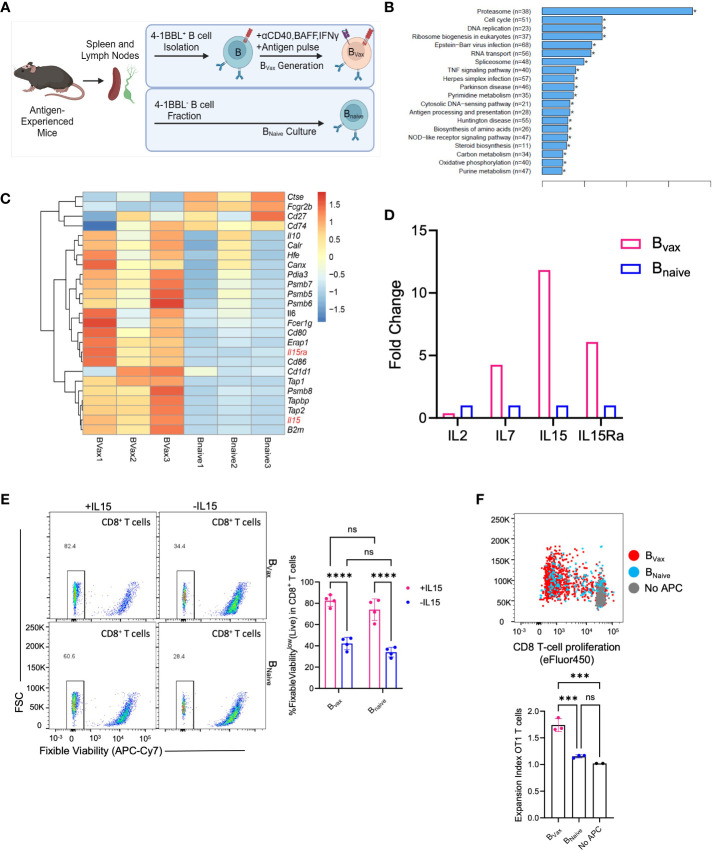
B_vax_ demonstrates superior antigen uptake and presentation function. **(A)** Illustration of B_Vax_ and B_Naive_ generation. **(B)** KEGG transcriptomic pathway analysis from bulk RNA sequencing comparing upregulated gene pathways in Bvax compared to B_Naive_. Of note, antigen processing and presentation pathway is upregulated in Bvax compared to B_Naive_. **(C)** Differential gene expression analysis between B_Vax_ and B_Naive_ with a curated gene list based on antigen uptake, processing and presentation gene sets. Heatmap was generated based on z scores calculated from FPKM. **(D)** Quantitative PCR analysis showing fold-change expression of IL2, IL7, IL15, and IL15Ra in B_Vax_ compared to B_Naive_. **(E)** CD8^+^ T cell viability after 3 days of culture with B_Vax_ or B_Naive_ with or without exogenous IL15. **(F)** Proliferation of OT1 T cells after co-cultured with B_Vax_, B_Naive_ or without APC (all supplemented with exogenous IL15) for 3 days. Histogram represented mean ± SEM. ns, p>0.05, ***p<0.001, ****p<0.0001.

With our spatial analysis of B and T cells in GBM suggesting that they physically occupy shared niches within the TME, we next wanted to evaluate functional interactions between B and T cells. Because naturally infiltrating B cells in GBM are suppressive cells that inhibit T cell expansion and function (5), we aimed to investigate alternative B cell subsets that could promote pro-inflammatory T cell activation. We previously described a B cell-based vaccine (B_Vax_) that had potent anti-glioma effect and can modulate the CD8^+^ T cell compartment *in-vivo*. To generate B_Vax_, 4-1BBL^+^ B cells from antigen experienced mice (either peptide immunized or tumor-bearing) were further activated with anti-CD40, B cell activating factor (BAFF), and IFNγ (**Figure 2A**).

Comparing the APC function of B_Vax_, bulk-RNA transcriptomic sequencing and GSEA analysis showed that B_Vax_ upregulated antigen processing and presentation pathways compared to naturally occurring naïve B cells (B_Naive_) (**Figure 2B**), which include the expression of genes such as *Tap1* and *Tap2* that form the transporter associated with antigen processing complex. Furthermore, bulk transcriptomic sequencing of B_Vax_ and B_Naive_ showed differences in cytokine expression, especially in the IL15 pathway, with upregulation of both IL15 and IL15Ra (**Figure 2C**) in the B_vax_ group. IL15Ra is critical for enhancing IL15 signaling by trans-presenting the cytokine to T cells (25, 26) which promotes the survival and homeostatic proliferation of memory CD8^+^ T cells (12, 27). Quantitative PCR analysis of IL15 and IL15Ra validated the bulk RNA sequencing results, with a 10-fold increase in IL15 gene expression and a 5-fold increase in IL15Ra gene expression in B_Vax_ versus B_Naive_ (**Figure 2D**).

To show the importance of the IL15-IL15Ra pathway in promoting T cell survival, CD8^+^ T cells were cultured with B_Vax_ or B_Naive_ with or without exogenous IL15 (50ng/mL). We found that addition of IL15 improved T cell survival after 3 days compared to T cells cultured without exogenous IL15, regardless of APC type (**Figure 2E**). We also found that coculture of T cells with B_Vax_ had improved survival compared to coculture with B_Naive_ when controlled for exogenous IL15, and that addition of IL15 to B_Vax_ promoted higher increase in T cell survival compared to the addition of IL15 to B_Naive_. CD8^+^ T cells cultured with 50ng/mL IL15 without any APC demonstrated nearly complete survival after 3 days along with evidence of non-specific T cell expansion (**Supplementary Figure 1A**), which had also previously been shown in the literature (28). By titrating the amount of IL15 we used to culture CD8^+^ T cells, we determined that 6.25 ng/mL was a suboptimal IL15 concentration that would promote cell survival without non-specific T cell expansion (**Supplementary Figure 1A**).

Next, we characterized antigen processing and cross-presentation using the OVA-SIINFEKL model by pulsing B_Vax_ or B_Naive_ with whole ovalbumin protein before culturing with OT1 transgenic CD8^+^ T cells (T cell receptors are specific for SIINFEKL, the MHC-I restricted OVA antigen) supplemented with exogenous IL15. APC to OT1 T cell ratio was 5 to 1. We observed that B_Vax_ can promote increased antigen-specific T cell expansion compared to B_Naive_ (**Figure 2F**). Our data suggest that B_Vax_ are potent antigen-presenting cells capable of higher levels of cross-presentation and the ability to expand antigen-specific CD8**^+^** T cells when compared to naïve B cells.

### B_Vax_ promotes CD8^+^ T cell expansion in murine brain tumors

Having shown that B_Vax_ are capable of antigen cross-presentation and expansion of antigen-specific CD8**^+^** T cells, we next looked to evaluate the ability of B_Vax_ derived from tumor-bearing mice to activate and expand CD8**^+^** T cells from tumor-bearing mice. DCs are the gold standard APCs, with DC-based vaccines being tested in GBM clinical trials (7, 8). Here, we use DCs generated from femoral bone marrow cells as a positive control for CD8^+^ T cell expansion. Briefly, B_Vax_ and DCs were generated from CT2A tumor-bearing mice and pulsed with CT2A tumor lysates. CD8**^+^** T cells were then isolated from CT2A tumor-bearing mice and cocultured with APCs supplemented with 6.25ng/ml of recombinant IL15 for 3 days.

**Figure 3:**
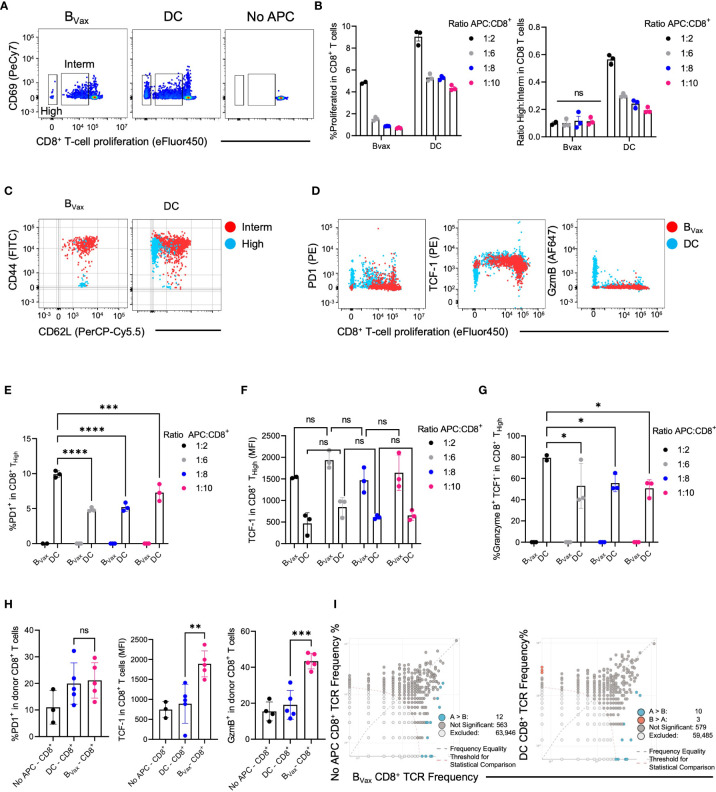
DC and B_Vax_ drive different T cell proliferation patterns *in vitro*. Proliferation dye-labeled B_Vax_- or DC-activated CD8+ T cells for 3 days with the addition of 6.25ng/ml IL15 on Day 0 and 48h post-co-culture. Abbreviations were used as High: highly proliferative cells; Interm: Intermediately proliferative cells. **(A)** *In-vitro* proliferation of CD8**^+^** T cells was measured by proliferation dye (eFluor450) dilution vs CD69 expression for B_Vax_, DC, B_Naive_, and no antigen-presenting cells (APC). Highly proliferative CD8**^+^** T cells were identified with the lowest proliferation dye expression. Intermediately differentiated CD8**^+^** T cells were the middle transitional population. No APC was used as baseline proliferation control. **(B)** Total proliferation (High + intermediately proliferative) in all CD8**^+^** T cells and the ratio of highly to intermediately proliferative CD8**^+^** T cells were plotted with mean ± SEM. APC were co-cultured with CD8**^+^** T cells at 1:2, 1:6, 1:8, and 1:10 ratios. **(C)** CD44 vs CD62L expression in intermediately differentiated (red) and highly differentiated (blue) CD8+ T cells. **(D)** Expression of PD-1, TCF-1, and Granzyme B in total CD8**^+^** T cell activated with B_Vax_ (red) or DC (blue) at 1:2 APC : CD8 ratio. **(E–G)** characterization of highly proliferative CD8**^+^** T cells by the expression of PD-1 **(E)**, TCF-1 **(F)**, and Granzyme B **(G)**. **(H)** After adoptively transferring 3M ex-vivo activated CD8**^+^** T cells to tumor-bearing Rag1 KO mice through an intracranial cannula, hosts’ brains were collected 3 days post-treatment for immunophenotyping. The expression of PD-1, TCF-1, and Granzyme B of the donor CD8**^+^** cells were shown in the histogram with mean ± SEM. ns, p>0.05, *p<0.05, **p<0.01, ***p<0.001, ****p<0.0001. **(I)** TCR clone overlaps from naïve (No APC), B_Vax_, and DC-activated CD8^+^ T cells TCR sequencing.

CD8**^+^** T cells cultured with different APCs had varying phenotypes after activation and expansion. B_Vax_ sequestered activated CD8^+^ T cells in an intermediately activated state. DCs promoted higher levels of total CD8^+^ T cell proliferation and increasingly pushed them to a highly proliferative state characterized by low expression of proliferation dye (**Figure 3A**; **Supplementary Figure 1D**). CD69 expression on proliferating T cells confirms TCR-mediated activation across APC groups (**Figure 3A**). The total amount of T cell proliferation depended on the density of APCs for both DCs and B_Vax._ DCs promoted more total CD8**^+^** T cell proliferation than B_Vax_ at the same APC density *in vitro* (**Figure 3B**). However, the ratio of highly proliferative T cells (High) to intermediately proliferative T cells (Interm) was not dose-dependent on B_Vax_ density. Still, it did vary based on DC density (**Figure 3B**).

To further characterize highly proliferative T cells from intermediately differentiated T cells, the expression of CD44 and CD62L was analyzed in both groups. CD44^+^CD62L^+^ T cells can be categorized as central memory T cells, whereas CD44^+^CD62L^-^ T cells are more effector T cells (9, 29). However, CD44^-^CD62L^+^ TCF-1^+^stem-like memory T cells are a unique subset of memory T cells with stronger proliferative potential than central or effector memory T cells (30, 31). IL15 monotherapy (6.25 ng/mL) did not induce T cell differentiation into CD44^+^CD62L^+^ T cells (**Supplementary Figures 1A–C**). We found that the highly proliferative group in B_Vax_-activated CD8**^+^** T cells consisted almost entirely of CD44^-^CD62L^+^ stem-like memory T cells.

In contrast, the intermediate group consisted entirely of CD44^+^CD62L^+^ central memory T cells (**Figure 3C**). Highly proliferative CD8 T cells in the DC group consisted mainly of CD44^+^CD62L^-^ effector T cells, with minimal presence of stem-like memory T cells. The intermediately proliferative CD8 T cells in the DC group also consisted mainly of central memory T cells but also had a large proportion of effector T cells (**Figure 3C**, **Supplementary Figure 1E**).

Moreover, we found that highly proliferative CD8**^+^** T cells in the B_Vax_ group did not express PD-1 or granzyme B (GzmB) but did express high levels of TCF-1, highly proliferative CD8**^+^** T cells in the DC group expressed high levels of PD-1 and granzyme B and lower levels of TCF1 (**Figures 3D, E, G**). To show that the generation of stem-like memory T cells in our B_Vax_-activated CD8**^+^** T cell group is due to a B_Vax_-specific property rather than APC density, we measured PD-1, TCF-1, and granzyme B expression in the highly proliferative T cells using different ratios of APC to CD8 T cells in the coculture. B cell density did not significantly affect PD-1, TCF-1, and granzyme B expression in highly and intermediately proliferative T cells. Decreasing DC density decreased the PD-1 and granzyme B expression in highly proliferative T cells but did not affect TCF-1 expression (**Figures 3E–G**; **Supplementary Figure F**).

To assess the antigen recall abilities of the *in-vitro* activated CD8**^+^** T cells, CD8**^+^** T cells were purified from our coculture system and adoptively transferred intracranially into tumor-bearing Rag knockout mice characterized as not harboring endogenous B or T cells. The B_Vax_-activated CD8^+^ T cells expressed similar levels of PD-1 as DC-activated CD8**^+^** T cells after adoptive transfer and antigen rechallenge but also maintained higher expression of TCF-1 and developed higher levels of granzyme B expression compared to DC-activated CD8**^+^** T cells (**Figure 3H**). TCR-sequencing further revealed that clones found in naïve CD8**^+^** T were also enriched in B_Vax_, and B_Vax_ expanded different TCR clones compared to DC. (**Figure 3I**).

Our data suggest that *in-vitro* activation of CD8**^+^** T cells with B_Vax_ promotes an expanded population expressing TCF1^+^, PD-1^-^, GZMB^-^, and CD44^+^CD62L^+^. These T cells are most consistent with previously described stem-like memory T cells that have unique TCR clones and can be reactivated *in-vivo* upon adoptive transfer.

### B_Vax_ promotes unique populations of highly and intermediately proliferative CD8^+^ T cells *in-vivo*

**Figure 4:**
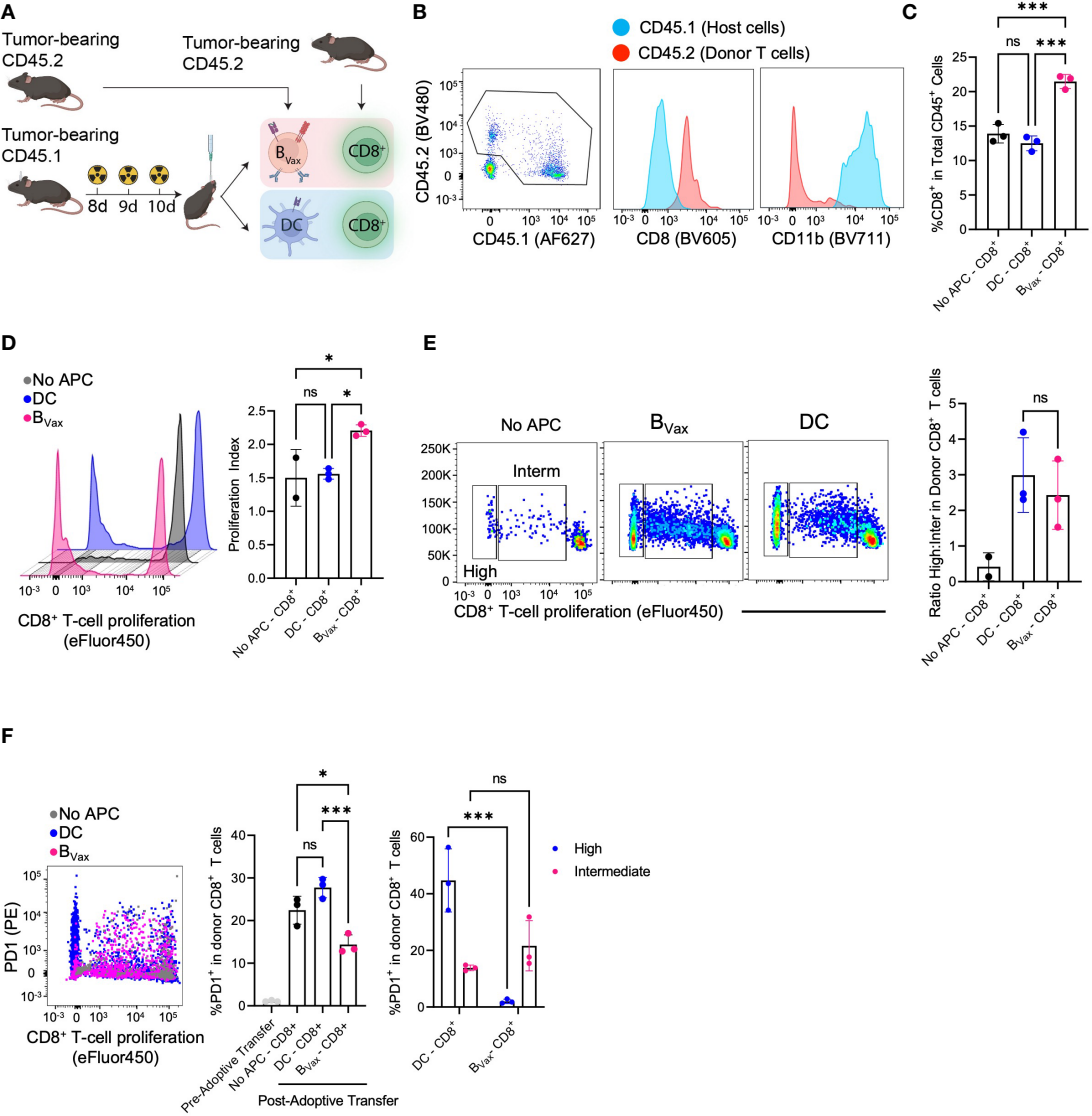
B_Vax_ expands stem-like CD8**^+^** T cell *in vivo*. **(A)** Schema of co-delivery APC and CD8**^+^** T cell and immunophenotyping. Briefly, CD45.1 host mice were implanted with 100K CT2A tumor cells through a cannula system and received a lymphodepleting dose of brain irradiation for 3 consecutive days. Co-delivery of 1.5M APC and 5M CD8**^+^** T cells from NUR-GFP^+^ (CD45.2) tumor-bearing donors. 3 days post-treatment, tumors were collected and processed for immunophenotyping. **(B)** Representative flow cytometry plot indicated tumor microenvironment (TME) mostly comprised of donor CD8**^+^** T cells (CD45.2) and host CD11b^+^ cells (CD45.1), whereas all the donor APC didn’t survive 3 days post treatment. **(C)** B_Vax_-activated CD8**^+^** T cells accumulated more than DC-activated CD8**^+^** T cells or No APC-CD8**^+^** T cells in the TME as quantified by the percentage of CD8 in CD45^+^ cells. **(D)** In a parallel experiment, CD8**^+^** T cells were labeled with CellTrace Violet before adoptive transfer into tumor-bearing mice. B_Vax_-activated CD8**^+^** T cells (pink) showed more total proliferation than DC-activated CD8+ T cells and no APC-CD8+ T cells as measured by loss of proliferation dye and proliferation. **(E)** Representative flow cytometry plot of T cell proliferation dye expression after adoptive transfer of CD8**^+^** T cells with B_Vax_, DC, or no APCs. The histogram shows the ratio of highly proliferative T cells to intermediately proliferative T cells in each of the 3 groups. **(F)** Flow cytometry measured the expression of PD-1 vs proliferation status of donor CD8+ T cells co-implanted with B_Vax_ (pink), DC (blue), and CD8+ T cell monotherapy. Pre-adoptive transfer CD8**^+^** T cells were freshly isolated from spleens and lymph nodes of tumor-bearing mice. Reduced PD-1 expression was observed in highly proliferative CD8 T cells co-implanted with B_Vax_. All histograms plotted mean ± SEM. ns, p>0.05, *p<0.05, ***p<0.001.

Having seen the different CD8**^+^** T cell phenotypes based on APC used to activate *in-vitro*, we sought to investigate the *in-vivo* effects of CD8**^+^** T cell activation using different APCs. Rather than coculturing CD8**^+^** T cells with APCs *in-vitro*, we developed an *in-vivo* system by adoptively transferring both the APC (CD45.2) and naïve CD8**^+^** T cell (CD45.1) from tumor-bearing mice into an immunocompetent tumor-bearing host that was treated with radiotherapy (**Figure 4A**). Donor CD8**^+^** T cells could be found in the brain three days after adoptive transfer and could be differentiated from host cells by CD45.1 expression (**Figure 4B**). Moreover, all CD8**^+^** T cells found in the brain were CD45.1 donor T cells, and host cells only comprised CD11b^+^ cells (**Figure 4B**).

Co-adoptive transfer of both B_Vax_ and CD8**^+^** T cells led to increased total CD8**^+^** T cells compared to co-adoptive transfer with DCs or no APCs (**Figure 4C**). Analysis of T cell proliferation showed that *in-vivo* B_Vax_ activation promoted increased CD8**^+^** T cell proliferation as measured by proliferation index (**Figure 4D**). Looking at proliferation cycles also allowed us to characterize activated CD8**^+^** T cells as highly or intermediately proliferative. *In-vivo* DC activation promoted an increased highly intermediately proliferative ratio compared to *in-vivo* B_Vax_ activation, similar to what we observed with our *in-vitro* coculture system (**Figure 4E**). However, like our *in-vitro* model, DC-activated CD8**^+^** T cells expressed higher PD-1 than B_Vax_-activated CD8**^+^** T cells (**Figure 4F**). Highly proliferative CD8**^+^** T cells in the DC group had significantly higher expression of PD-1 compared to intermediately proliferative CD8**^+^** T cells. This difference was not observed in comparing the high vs intermediately proliferative CD8 T cells in the B_Vax_ group (**Figure 4F**).

Thus, our study shows that B_Vax_ are powerful APCs capable of promoting T cell activation and proliferation *in-vivo* in the brain tumor microenvironment. Such *in-vivo* B_Vax_-mediated T cell activation resembles a similar phenotype as *in-vitro* B_Vax_ activation.

## Discussion

Our study shows that B and CD8**^+^** T cells can co-localize in the GBM tumor microenvironment. This suggests that immunological synapse formation between B cells and T cells in GBM is spatially feasible. Because we showed previously that B cells that migrate to the TME become immunosuppressive (5), we sought to lay the groundwork for investigating using B_Vax_ to overcome TME immunosuppression and modulate CD8**^+^** T cell function. To better understand the functionality of B_Vax_ and T cell interactions, we characterized B_Vax_ antigen presentation capability. We found that B_Vax_ are more potent antigen-presenting cells compared to naïve B cells, with increased expression of antigen processing gene pathways and surface epitope presentation. We also found that B_Vax_ has elevated IL15 signaling pathway compared to naïve B cells. B_Vax_’s unique APC profile was also reflected in its ability to generate stem-like memory CD8**^+^** T cells both *in-vitro* and *in-vivo*, which DCs could not do in GBM. These stem-like memory CD8**^+^** T cells are characterized by elevated TCF1 expression and CD62L^+^CD44^-^ throughout multiple activation and proliferation cycles and were able to further proliferate after an *in-vivo* antigen rechallenge.

The first important point of our study is that despite GBM being an immunologically cold tumor, we can still visualize B and T cell co-localization within distance to form immunological synapses. Our new study adds improved granularity to the clustering of different immune cells as they relate to B cells. Based on our histological and spatial data, it is likely that clustering of B cells and CD8**^+^** T cells occur mainly around vessels. While our study does not directly investigate tertiary lymphoid structure formation, improving B cell functionality in the TME can improve the likelihood of TLS formation and thus improve immunotherapy efficacy. In our spatial analysis, we can see areas of B and T cell colocalization with myeloid cells surrounding what resembles the formation of early lymphoid structures with direct interactions between B and T cells. Without any immunotherapy to either block myeloid-mediated immunosuppression or enhance lymphocyte immunity, it is possible that these myeloid cells surround these structures and inhibit any immune activation that could be happening otherwise.

The second goal of our study was to better show how different APCs can lead to different T cell phenotypes after activation. B_Vax_ is a unique set of B cells generated from the costimulatory 4-1BBL^+^ B cells isolated from a tumor-bearing host. These cells have already undergone B cell receptor-mediated activation and have anti-tumor specificity. While investigations of CD8**^+^** T cell activation have long focused on DCs as the gold standard of antigen presentation, an increasing body of work points to several factors that make B cells powerful APCs, such as the ability to share cognate antigen-specificity with T cells, improved circulatory mobility, and unique cytokine expression profiles (6). We show that B_Vax_ and DCs generate different subsets of activated CD8**^+^** T cells, with DCs promoting rapid effector differentiation. Our data showed that these DC-generated effector T cells can potentially face rapid exhaustion and immunosuppression and have less proliferation potential after adoptive transfer into the TME.

Our study used IL15 to promote T cell expansion based on RNA sequencing data suggesting Bvax upregulate IL15Ra compared to naïve B cells. IL15 pathway has been previously shown to help drive T cell memory differentiation (32), but our paper suggests that the APC used to activate the CD8 T cells also affects the final T cell phenotype, as Bnaive, Bvax, and DCs all with exogenous IL15 produce different T cell phenotypes. We speculate that Bvax utilizes IL15 to activate the Wnt pathway, as the Wnt pathway has been shown to drive stem-like memory differentiation in T cells (33). It could also be possible that Bvax utilizes a different signaling pathway such as 4-1BB/4-1BBL signaling (34) that drives memory T cell differentiation.

Our study has several important clinical implications. Reports are now showing how different subsets of CD8**^+^** T cells based on expression of TCF-1 impact immunotherapy response (18). While the exact mechanism at play serves as a point of future investigation, we show a role for B_Vax_ as an important tool to generate TCF-1^+^ stem-like memory T cells *in-vitro*. Because our data show that B_Vax_ can continue to activate CD8**^+^** T cells *in-vivo*, we envision a dual role for both B_Vax_ and DC vaccines where B_Vax_ serves to maintain a pool of anti-tumor stem-like T cells, and DC vaccines rapidly generate effector cytotoxic T cells to kill the primary tumor or any recurrence.

## Data availability statement

The T cell RNA-Seq dataset presented in the study is deposited in the NCBI Sequence Read Archive repository, accession number PRJNA1047925; The B cell RNA-Seq and TCR-seq dataset presented in this article are not readily available because of the loss of raw files. Requests to access the datasets should be directed to CL; catalina.leechang@northwestern.edu.

## Ethics statement

Approval for collection was granted under the institutional review board protocol number STU00202003 and the study was conducted following the U.S. Common Rule of ethical standards. The studies were conducted in accordance with the local legislation and institutional requirements. The participants provided their written informed consent to participate in this study. The animal study was approved by Institutional Animal Care and Use Committee at Northwestern University under the protocol number ISO16696. The study was conducted in accordance with the local legislation and institutional requirements.

## Author contributions

DH: Conceptualization, Data curation, Formal analysis, Investigation, Methodology, Project administration, Software, Supervision, Validation, Writing – original draft, Writing – review & editing. HW: Conceptualization, Data curation, Formal analysis, Investigation, Methodology, Project administration, Software, Supervision, Validation, Writing – original draft, Writing – review & editing. JK: Data curation, Investigation, Project administration, Writing – review & editing. SW: Data curation, Formal analysis, Investigation, Methodology, Writing – review & editing. BC: Data curation, Formal analysis, Investigation, Methodology, Writing – review & editing. GV: Data curation, Investigation, Methodology, Writing – review & editing. VA: Data curation, Formal analysis, Investigation, Methodology, Validation, Writing – review & editing. SD: Data curation, Formal analysis, Investigation, Writing – review & editing. HN: Data curation, Methodology, Formal analysis, Validation, Investigation, Writing – review & editing. AR: Data curation, Formal analysis, Investigation, Writing – review & editing. TC: Data curation, Investigation, Writing – review & editing. TA: Data curation, Investigation, Writing – review & editing. JC: Data curation, Investigation, Writing – review & editing. LB: Data curation, Investigation, Writing – review & editing. AL: Project administration, Writing – review & editing. YH: Methodology, Project administration, Writing – review & editing. AS: Data curation, Supervision, Writing – review & editing. AH: Data curation, Supervision, Validation, Investigation, Writing – review & editing. PZ: Data curation, Writing – review & editing. JM: Conceptualization, Formal analysis, Investigation, Resources, Supervision, Visualization, Writing – original draft, Writing – review & editing. CL: Conceptualization, Data curation, Formal analysis, Funding acquisition, Investigation, Methodology, Project administration, Resources, Software, Supervision, Validation, Visualization, Writing – original draft, Writing – review & editing.

## Supplementary material

Supplementary Figure 1
**(A–C)** Viable number, memory phenotype differentiation, and PD-1 expression of 1M CD8^+^ T cells cultured with varying concentrations of IL15 from 0.39ng/ml to 50ng/ml for 72h. 6.25ng/ml of IL15 didn’t significantly promote CD8^+^ T cell proliferation compared to 20ng/ml IL2 **(A)** but hampered central memory differentiation **(B)** and PD-1 expression **(C)**. **(D)** Gating strategy for proliferating CD8^+^ T cells. **(E)** Quantified data of CD62L CD44 expression in intermediately and highly proliferative CD8**^+^** T cells activated by B_Vax_ or DC at different APC densities. **(F)** Quantified PD-1 and TCF-1 expression data in intermediate CD8^+^ T cells at different APC densities. **(G)** Differential gene expression analysis between B_Vax_ and DC-activated CD8^+^ T cells. All histograms plotted mean ± SEM. ns= p>0.05, * = p<0.05, *** = p<0.001.
