# Supplementary figures and images for: Antigen-presenting B cells promote TCF-1+ PD1- stem-like CD8+ T-cell proliferation in glioblastoma
Source: Front Immunol. 2024 Jan 10;14:1295218. doi: 10.3389/fimmu.2023.1295218 (PMC10806106; doi:10.3389/fimmu.2023.1295218)

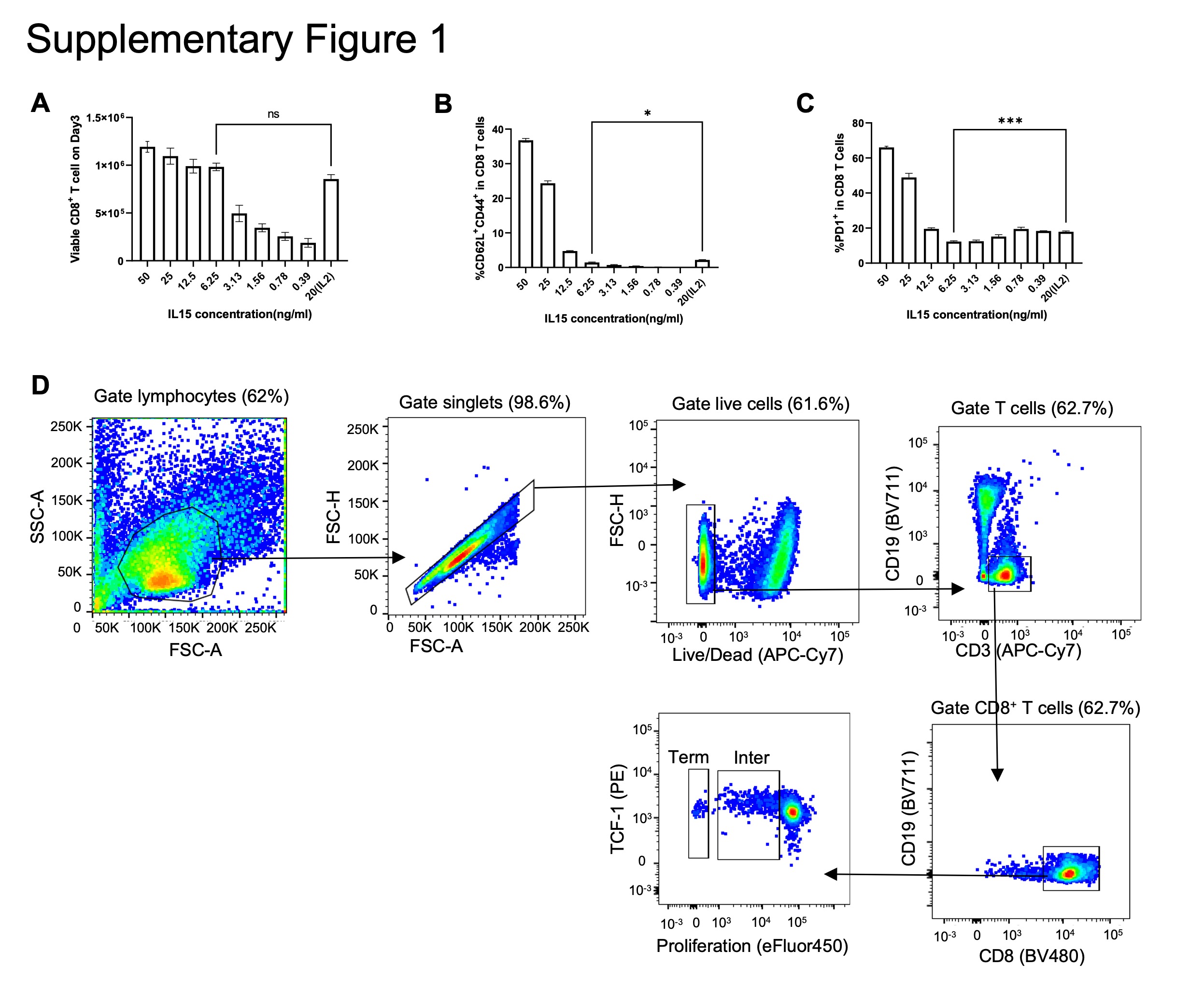

Supplement: Supplementary Figure 1 — (A–C) Viable number, memory phenotype differentiation, and PD-1 expression of 1M CD8+ T cells cultured with varying concentrations of IL15 from 0.39ng/ml to 50ng/ml for 72h. 6.25ng/ml of IL15 didn’t significantly promote CD8+ T cell proliferation compared to 20ng/ml IL2 (A) but hampered central memory differentiation (B) and PD-1 expression (C). (D) Gating strategy for proliferating CD8+ T cells. (E) Quantified data of CD62L CD44 expression in intermediately and highly proliferative CD8 + T cells activated by BVax or DC at different APC densities. (F) Quantified PD-1 and TCF-1 expression data in intermediate CD8+ T cells at different APC densities. (G) Differential gene expression analysis between BVax and DC-activated CD8+ T cells. All histograms plotted mean ± SEM. ns= p>0.05, * = p<0.05, *** = p<0.001. [file Image_1.jpeg]
